# Empowering future nurses: enhancing self-efficacy, satisfaction, and academic achievement through talent management educational intervention

**DOI:** 10.1186/s12912-025-03512-z

**Published:** 2025-07-07

**Authors:** Fahad M. Alhowaymel, Manal Saleh Moustafa Saleh, Nagwa Nabeh Taref, Zaineb Naiem Abd-Elhamid, Abdulaziz F. Abaoud, Atallah Alenezi, Riham Hashem Fathi, Hanan El Said Elsabahy

**Affiliations:** 1 Department of Nursing Sciences, College of Applied Medical Science, Shaqra University, Shaqra, Saudi Arabia; 2 Nursing Administration, Faculty of Nursing, Zagazig University, Zagazig, Egypt; 3 Community Health Nursing, Faculty of Nursing, Mansoura University, Mansoura, Egypt; 4 College of Nursing, Imam Mohammad Ibn Saud Islamic University (IMSIU), Riyadh, Saudi Arabia; 5 Faculty of Medicine, Zagazig University, Zagazig, Egypt; 6 Nursing Administration, Faculty of Nursing, Mansoura University, Mansoura, Egypt

**Keywords:** Talent management, Nursing students’ self-efficacy, Satisfaction, Academic achievement

## Abstract

**Background:**

Nursing education plays a pivotal role in shaping the competence and confidence of future healthcare professionals. As the demands and complexities of healthcare delivery continue to evolve, there is an increasing need to explore innovative approaches to empower nursing students and enhance their self-efficacy, job satisfaction, and academic achievement.

**Objectives:**

To enhance self-efficacy, satisfaction, and academic achievement among future nurses through talent management educational intervention.

**Methods:**

A quasi-experimental design was employed, utilizing a pre-test and post-test identified through a systematic random sampling method. Nursing students were equally distributed between the study and control groups (*N* = 93, each). The time of data collection was between August and November 2023. Five tools were used to measure the following areas: Talent Management Knowledge Questionnaire (TMKQ), Talent Management Competency Scale, Students’ Self-Efficacy Questionnaire, Students’ Satisfaction Questionnaire, Students’ Academic Achievement Questionnaire. The study used SPSS for statistical analyses, assessing data normality using a one-sample Kolmogorov-Smirnov test. Data was expressed as mean and standard deviation, with categorical data as numbers and percentages. Chi-square tests were used for comparison. Independent and paired t-tests were used for comparison. A correlation coefficient test was used to assess correlations.

**Results:**

The findings of this study demonstrate that the talent management educational intervention significantly enhanced nursing students’ outcomes across multiple domains. Post-intervention results revealed substantial improvements in students’ knowledge, talent management competencies (including attraction, development, and retention), self-efficacy, satisfaction, and academic achievement compared to the control group. The intervention led to marked increases in mean scores, with knowledge rising from 38.7 to 81.7, and self-efficacy from 13.9 ± 2.0 to 19.1 ± 1.7. Additionally, high levels of satisfaction (82.8%) and academic achievement (74.2%) were reported. A highly significant positive correlation was observed between students’ knowledge, talent management competency, self-efficacy, satisfaction, and academic performance (*P* ≤ 0.0001), indicating the effectiveness of the intervention in empowering future nurses.

**Conclusions:**

The talent management educational intervention proved to be a highly effective strategy for enhancing nursing students’ outcomes across a spectrum of critical domains. The substantial gains observed in knowledge, talent management competencies, self-efficacy, satisfaction, and academic achievement, coupled with the highly significant positive correlations between these factors, underscore the intervention’s success in empowering future nurses with essential skills and fostering a positive learning experience. These findings strongly advocate for the integration of talent management principles into nursing education to cultivate well-prepared and confident nursing professionals.

**Clinical trial number:**

Not applicable.

**Supplementary Information:**

The online version contains supplementary material available at 10.1186/s12912-025-03512-z.

## Introduction

In healthcare, nurses and nurse managers play a pivotal role in providing compassionate care, promoting health, and supporting patients through their most vulnerable moments. As the demands on healthcare systems continue to evolve, the significance of competent and empowered nursing professionals becomes increasingly evident [1]. The professional nurses and head nurses of the future, who will work alongside colleagues and other healthcare professionals to provide health and educational services to patients, their families, and the community, are developed from the foundation established by today’s nursing students [2]. In this era of constant change, talent management is crucial for enhancing human capital value. Emphasis should be placed on employee selection, talent development, and limiting competency loss, particularly in educational institutions like universities and schools [3]. Central to the cultivation of a robust nursing workforce is not only the acquisition of clinical skills and knowledge but also the nurturing of attributes such as self-efficacy, job satisfaction, and academic achievement [4].

Nursing students’ self-efficacy and readiness for future clinical roles, particularly in hospital settings, are greatly impacted by the absence of empowerment training. Empowerment training is essential because it gives students the self-assurance and abilities they need to handle the challenges of clinical decision-making and patient care. Without this kind of instruction, nursing students might not be able to function well under pressure, which could cause them to become more anxious and have less job satisfaction when they start working. Additionally, academic success may be hampered by educational institutions’ lack of formal talent management techniques. Finding and developing students’ talents is part of talent management, which can improve learning outcomes and experiences for students, which will ultimately lead to improved performance in clinical settings. Effective talent management strategies have been linked to academic success in studies, indicating that when nursing programs support their students’ potential development, they not only increase self-efficacy but also equip them for the demands of their future careers in healthcare [5].

This research aims to explore a new strategy for developing key attributes in aspiring nurses through an educational intervention in talent management. Talent management, commonly associated with corporate settings, involves attracting, developing, and retaining skilled individuals [6]. Applying these concepts to nursing education holds the potential for producing proficient and satisfied nursing professionals [7]. As a strategic tool, talent management can provide organizations with a lasting competitive edge. Over the past decade, rapid changes in accreditation, learning outcomes, and quality have required educational institutions, especially in nursing, to prioritize talent management and develop highly skilled students to remain competitive [8].

Talent management involves combining tactical thinking, leadership, and communication skills with the ability to motivate and attract talented students. It focuses on efficiently attracting, recognizing, and utilizing these students for organizational success, guiding institutions and universities toward healthier talent creation [9]. Talented students are viewed as valuable assets to society, especially in the pursuit of progress and development. With unique abilities and skills, they think creatively and solve problems effectively. Educational institutions aim to identify these students and design special programs to nurture and utilize their talents [10].

The primary focus of this intervention is to bolster self-efficacy, defined as an individual’s belief in their capacity to execute behaviors necessary to produce specific performance attainments [11]. Research indicates that higher levels of self-efficacy among nursing students are associated with improved academic performance, clinical competence, and resilience in the face of challenges [12]. By instilling confidence in their abilities, nursing students are better equipped to navigate the rigors of their education and future professional endeavors. Prior research has also revealed significant direct effects of students’ self-efficacy on academic expectations [13].

Competent self-efficacy is the belief in one’s ability to perform tasks and achieve desired outcomes. For nursing students, developing self-efficacy is crucial to becoming competent and independent, leading to successful training and a rewarding nursing career [14]. Nurses’ self-efficacy and competence are linked to improved professional skills, academic success, patient safety, and healthcare outcomes [15]. In an academic setting, self-efficacy is essential for proficient nursing students to perform clinical tasks independently and reflects the workplace’s commitment to their roles [16].

This intervention seeks to address the issue of satisfaction among nursing students, a factor intricately linked to retention rates within the profession. Dissatisfaction during the formative years of nursing education can precipitate attrition and contribute to workforce shortages [17]. By proactively attending to the factors influencing job satisfaction, such as supportive learning environments, mentorship opportunities, and recognition of accomplishments, educators can lay the groundwork for sustained engagement and commitment among their students [18, 19].

Satisfaction among nursing students plays a crucial role in their academic and professional development [20]. High levels of satisfaction are often linked to better academic performance, enhanced motivation, and a stronger commitment to the nursing profession. Additionally, fostering leadership and managerial skills in nursing students is essential for preparing them to take on more responsible roles in healthcare settings. Developing these skills helps students navigate complex healthcare environments, improve decision-making, and contribute to better patient outcomes while positioning them for future leadership positions. [21, 22].

The intervention aims to augment academic achievement by offering tailored support that aligns with individual learning needs and preferences [7]. Recognizing that diverse learners require different instructional approaches, the intervention fosters an inclusive environment that supports various learning styles, promoting belonging and academic success [23]. Academic achievement reflects the attainment of educational goals by learners, instructors, or institutions [11]. Research links emotional intelligence and self-efficacy to academic success, with positive outcomes achieved through resources, safe practice areas, and supportive environments [4]. Even though self-efficacy, satisfaction, and academic accomplishment among nursing students have been studied globally, talent management practice programs for nursing students have not been studied in conjunction with these characteristics. Also, graduates must possess strong interpersonal skills and talent to avoid negative professional attitudes and behaviors. This will help to promote competent self-efficacy and safe patient care [24]. Furthermore, according to [25], contented students are more successful and committed to achieving their goals than dissatisfied students. Similar to this, gifted students assist in forming new friendships and promote their university to outsiders [10]. Lacking students were more likely to develop psychological distress during their studies and to drop out of college. So, there is a need to practice talent management programs on nursing students and affect self-efficacy competency, satisfaction, and academic achievement.

## Theoretical framework

### Social cognitive theory (Albert Bandura)

Bandura (1986) [26] created the Social Cognitive Theory (SCT), which offers a fundamental perspective for comprehending how learning and development are influenced by the interaction of cognitive, behavioral, and environmental factors. The idea of self-efficacy, or a person’s confidence in their ability to carry out the actions required to achieve particular performance outcomes, is fundamental to SCT [27]. In nursing education, increasing students’ self-efficacy is essential for boosting academic achievement, boosting clinical abilities, and fostering confidence. Self-efficacy can be considerably increased by talent management educational programs that incorporate goal-setting, skill development, mentoring, and constructive criticism [28]. As a result, this study uses SCT to clarify how these interventions empower aspiring nurses by enhancing their motivation and providing them with competencies.

### Self-Determination theory (Edward deci & Richard Ryan)

Deci and Ryan’s self-determination theory (SDT) focuses on the intrinsic motivation that propels people to develop and achieve as long as three basic psychological needs—autonomy, competence, and relatedness—are satisfied [29]. Academic success and student satisfaction can be greatly increased in nursing education through interventions that respect students’ autonomy, increase their sense of competence through skill training, and cultivate meaningful relationships through mentorship and collaborative learning environments [30]. Because they provide individualized, structured growth opportunities that encourage internal motivation, talent management educational interventions are very compatible with SDT principles. Fostering intrinsic motivation in healthcare students has been shown to improve academic performance as well as long-term professional engagement and retention [31].

In essence, this paper proposes an innovative paradigm for nurturing the next generation of nursing professionals by integrating talent management principles into educational practices. By enhancing self-efficacy, job satisfaction, and academic achievement among nursing students, this intervention endeavors to fortify the foundation upon which a resilient and proficient nursing workforce is built. Through empirical investigation and critical analysis, this study seeks to elucidate the efficacy and implications of such an approach, offering valuable insights for nursing education and practice alike.

### Aim of the study

#### Primary objective

To enhance self-efficacy, satisfaction, and academic achievement among future nurses through talent management educational intervention.

#### Secondary objectives

1. To identify the specific areas of talent management that can positively impact self-efficacy among future nurses.
2. To assess the correlation between satisfaction levels and the implementation of talent management strategies in nursing education.
3. To assess the academic achievement improvements resulting from talent management educational intervention among future nurses.

### Hypotheses of the study

#### H1

There will be a significant difference between pre- and post-educational intervention regarding knowledge of talent management among the study group.

#### H2

pre-educational and post-educational intervention will have a significant difference in self-efficacy, satisfaction, and academic achievements among future nurses.

#### H3

There will be a significant difference between the study and control group regarding self-efficacy, satisfaction, and academic achievements.

## Methods

### Study design

The study’s design was quasi-experimental. The researchers conducted pre-and post-tests to examine the objectives of the study between the control and study groups.

### Study setting and participants

In a Saudi Arabian governmental university, undergraduate nursing students participated in the current study. The study included a sample of 186 nursing students from the Bachelor of Science in Nursing program at all levels were equally distributed between the study group and a control group (*N* = 93, each). The participants were students, both male and female, who were proportionally selected based on program level (i.e., 2nd (0.50), 3rd (0.32), and 4th (0.18). They were evenly assigned to the control and study groups (*N* = 93, each). In order to guarantee a wide variety of experiences and viewpoints in the study, nursing students in their second, third, and fourth levels of education were specifically chosen. This diversity enables a more thorough comprehension of the intervention’s efficacy at various training levels. We used a uniform training approach and gave each participant similar access to resources in order to lessen the possible detrimental effects of different experience levels on the intervention. By keeping the learning environment consistent, this strategy made sure that variations in knowledge or proficiency wouldn’t distort the outcomes. In order to promote peer support, we also led group discussions where less experienced students might gain knowledge from their more seasoned peers.

A systematic random sampling procedure was employed to identify participants to get the required sample size of nursing students. The Epi website (Open-Source Statistics for Public Health) was utilized to determine the necessary sample size to assess the intervention’s efficacy, using the formula,

$$\begin{aligned} \:\left[\text{D}\text{E}\text{F}\text{F}\text{*}\text{N}\text{p}\:\right(1-\text{p}\left)\right]&=\text{n}/\:[/2\text{*}(\text{N}-1)\hspace{0.17em}\cr&+\text{p}\text{*}(1-\text{p}\left)\right]\:\text{d}2/\text{Z}2\end{aligned} $$

Where N and n stand for the population and sample sizes, respectively, and P=% frequency of knowledge among nursing students = Confidence limits as % of 100 (absolute +/- %) (d) = 5%, Z = 1.96, and α = 0.05. One of the two criteria entered was Odd’s Ratio (OR), which would be calculated by the Epi website program. The alternative estimate, which came from pilot research, was % of exposed) = 10%. The equation solutions were presented using the Fleiss method [32].

### Inclusion and exclusion criteria

According to the study’s inclusion requirements, participants must be enrolled in a nursing program at the designated institution and be in their second, third, or fourth levels, with a proportionate representation from each level. To secure legal consent, participants must be at least 18 years old and free from serious health conditions that could limit their ability to participate or influence the results. On the other hand, in order to guarantee sufficient exposure to nursing education, the exclusion criteria state that students who are not majoring in nursing or who have not finished at least one semester of the nursing program are not eligible. In order to prevent bias, volunteers who have taken part in comparable studies in the last 12 months will also be disqualified, as would those who are unable to understand the study.

### Data collection

Five instruments were utilized in this study to collect data namely: Talent Management Knowledge Questionnaire (TMKQ), Talent management competency Scale, Students’ Self-Efficacy Questionnaire, Students’ satisfaction questionnaire, Students’ academic achievement questionnaire. The Talent Management Knowledge Questionnaire (TMKQ) is one tool that the researchers created for this study. The Arabic version of the TMKQ was later translated from its original English version. Using the technique of translation and back-translation, the other tools were likewise translated into Arabic [33]. Further details about the translation processes and information about reliability and validity are provided below.

### Study measures

#### **Tool (1): talent management knowledge questionnaire (TMKQ)**were included two parts

##### Part (1) Demographical characteristics

The information about demographical characteristics that were collected from participating students included the following: age, gender, academic year, marital status, and work besides studying and students entering the nursing department on a personal desire.

##### Part (2) TMKQ questionnaire

 was developed by the researchers for this study based on the previous studies and literature [34, 35]& [36] (Mohammed et al. 2017; Yener et al. 2017 Mujtaba, 2022). This instrument included 27 questions to evaluate nurse students’ knowledge about talent management before and after educational intervention. The 27 questions are classified as a series of multiple-choice (7 items), and true & false questions (20 items) to be implemented in every session of the program. (Supplementary File − S1).

###### Scoring system

knowledge items were measured using a binary response (0 or 1) for each of the 27 questions, where 0 refers to an incorrect answer, and 1 refers to a correct answer. Then, the researchers summed up all answers yielding a total score for the TMKQ in the range (0–27). Consistent with [36], Mujtaba, (2022) the total score of each studied nursing student has been categorized arbitrarily into “inadequate knowledge” when the student achieved less than 60% (0 – <16.2) of the total score, “and “adequate knowledge” when the student achieved equal or more than 60% (≥ 16.2) of the total score.

#### Tool (2): talent management competency scale

The talent Management Competency Scale firstly, was developed by [37] Oehley, (2007) and the scale consists of 8 dimensions and 43 items were factors loadings generally satisfactory, varying between 0,569 and 0,895 with a mean of 0,755 and a median of 0,762. Secondly, the scale adopted for talent management competency among nurses by [38] El Dahshan et al., (2018) is based on [39] El Nakhala (2013) and the scale consists of (3) dimensions and thirty-one items with Cronbach’s coefficient alpha (0.84).

Thirdly, this tool was adopted from [38] El Dahshan et al., (2018) and aimed to examine talent management competency among nursing students were tool included 31 items representing the three theoretical aspects of talent management competency; namely, attracting talent (10 items) e.g.: the college can attract students even though the limited supply of skilled and talented students; talent development (10 items) e.g.: the collage encourages talented students to develop their skills, and talent retention (11 items) e.g.: the benefits at the college are fair and consistent. Talent management competency items were measured using a 5-point Likert scale (1–5), where 1 refers to never satisfied and 5 refers to highly satisfied. Based on [38] El Dahshan et al., (2018) a 50% cut-off point, talent management competency for nursing students total scores were divided into three categories: low (< 50%), moderate (50–75%), and high (> 75%).

#### Tool (3): students’ self-efficacy questionnaire

The students’ self-efficacy questionnaire was developed by [40] Glynn et al., (2011). This questionnaire aimed to assess nursing students’ Self-efficacy levels, consisting of five questions (e.g.: I expect to do as well as or better than other students in the nursing courses). The students’ self-efficacy questions were measured using a 4-point Likert scale (1–4), where 1 refers to never and 4 refers to always. The total scores were categorized into three categories: low self-efficacy (< 60%), moderate self-efficacy (60 – < 80%) and high self-efficacy (≥ 80%). The higher the score means the greater the student’s self-efficacy according to [40], Glynn et al., (2011).

#### Tool (4): students’ satisfaction questionnaire

This questionnaire was developed by [41] Clemes et al. (2008) to assess the degree of satisfaction among nursing students. The questionnaire included 39 questions, divided into three categories; namely, satisfaction with faculty staff members, support staff, and employees made up of twenty questions (e.g.: Faculty staff is polite and courteous, etc.), satisfaction with the physical environment consisted of nine questions (e.g.: The library has an extensive collection of available books and periodicals, etc.), and satisfaction with the learning courses used in the faculty contained ten questions( e.g.: As a result of attending the learning courses I have acquired a broad general education in different fields, etc.). The students’ satisfaction questions were measured using a 4-point Likert scale (1–4), where 1 refers to never and 4 refers to always. Next, the overall score of the nursing student was determined, multiplied by 100, and converted into a percent. Then divided into the following three categories: low satisfaction < 60% (0–93.6), moderate satisfaction 60 – < 80% (93.6 – < 124.8), and high satisfaction ≥ 80% (≥ 124.8). The higher the score means the greater the student’s satisfaction according to [41], Clemes et al. (2008).

#### Tool (5): students’ academic achievement questionnaire

This questionnaire was developed by [42] Zimmerman and Kitsantas (2014) and modified by [43] Alenezi et al., (2020). This questionnaire consisted of 45 self-report statements to capture respondents’ views of their academic achievement. The tool was divided into five main categories; namely, academic performance (e.g.: Studying hard to understand the content thoroughly.), extracurricular activities (e.g.: Participating in extracurricular events (sports, clubs)., student interaction (e.g.: Participating in class discussion.), student’s behavior (e.g.: Listening carefully during a lecture on a difficult topic.), and student’s attendance (e.g.: Attending class regularly.) Each category has nine statements.

The student’s academic achievement items were measured using a 5-point-Likert scale (0–4), where 0 refers to very little efficacy and 4 refers to quite a lot of efficacies. The scores for each category were summed to give a total score and categorized into three categories: lower academic achievement (0 – <60), moderate academic achievement (60 – <120), and higher academic achievement (120–180). The higher score means the greater the student’s academic achievement.

### Translation procedures, pilot study, and the tool’s validity and reliability

As the original language of participating students is Arabic and to ensure full understanding of the questions, all study measures were translated into the Arabic language. The translation was reviewed by a panel of five university professors, all are bilanguage of both languages (i.e., English and Arabic). Furthermore, another panel of five university professors examined the measures back translation from Arabic to English. Ten of the nursing professors who participated in the reviewing process of translation examined the content and face validity of the measures. The panelists reported that measures are valid to use in this study, and nothing requires modifications based on the panel’s recommendations. The Factor loadings were acceptable, and confirmed to be above 0.40, supporting the knowledge questionnaire’s dimensional structure.

Furthermore, an initial pilot study was conducted on 18 (10%) participants of the study population. The pilot study evaluated the study instruments’ readability, applicability, and time demand as well as their viability. The study results remained unchanged after the pilot study’s findings were integrated.

Reliability was estimated among 18 nursing students using the test-retest method two weeks apart. Then Cronbach alpha reliability test was done using the SPSS software. Cronbach’s alpha coefficients were calculated for multipoint items to evaluate the measurement reliability. Cronbach’s alpha for each tool was as follows: 0.871 for the first tool (TMKQ questionnaire), 0.901 for the second tool (Students’ Talent management competency), 0.897for the third tool (Students’ self-efficacy), 0.903 for the fourth tool (Students’ satisfaction), and 0.899 for the fifth tool (Students’ academic achievement). The results of the Cronbach’s alpha reliability test for the five tools indicated that they were reliable in detecting the objectives of the study.

### Study procedure (talent management educational intervention - (Table 1 )

Talent management educational intervention included the following phases:

#### Phase one: interview with nursing students and assessment

After receiving the permission, the study’s purpose and the approach to completing the questionnaires were clearly explained to all participants. The researcher met with them at different grade levels based on their schedules and distributed the study tools to them. To gather information about the nurse students' all the tools distributed to all the nurse students before the educational intervention started.

#### Phase two: the implementation of the talent management educational intervention

The training program was implemented during the first academic semester in the time between August and November 2023. Participating Students were assigned to seven groups, each group of nursing students included (23–26) students. Each group trained for two weeks, with four sessions per week, and each session was two hours and a half.

The program was implemented for nursing students in conference rooms in the faculty. Additionally, the following teaching methods were used: brainstorming, discussion, working in small groups, and modified lectures on talent management, satisfaction, self-efficacy, and student achievement effectiveness.

The length of the class and the promotion of attendance are important elements intended to improve learning outcomes in the context of a talent management educational intervention for nursing students. Usually lasting two to three hours, these classes are designed to be participatory and interesting in order to keep students’ attention and maximize their recollection of the material. Although some students might worry that these extra sessions will interfere with their usual academics, the intervention is planned to enhance rather than replace their current curriculum. The program seeks to establish a sense of community and accountability among students by creating a supportive atmosphere that prioritizes attendance. This will ultimately improve students’ professional growth without taking away from their academic obligations. This balance is essential in ensuring that students can integrate the skills and knowledge gained from the intervention into their regular studies and clinical practice.

**Table 1:** Talent management training sessions, goals, and activities

Sessions	Goals	Activities
**First session** (Theoretical background)	1. Establish the group 2. Introduce the program 3. Introduce the concept of talent management. 4. Principles and benefits of talent management. 5. Explore the importance of talent management. 6. The categories and effectiveness of talent management were discussed as well as the barriers to sustaining talent management.	(i) Warm-up game and acquaintances (ii) Establishment of a group contract (iii) Clarification of the program’s aim, activities, and timeline (iv) Explanation of the concepts of talent management by the researcher (v) Explore the Principles, benefits and importance of talent management. (vi) Description of the categories and effectiveness of talent management was discussed as well as the barriers to sustaining talent management
**Second session** (model and clarify the successful talent management)	1. Discuss the communication and motivation in talent management 2. Discuss the model of talent management 3. Focus on the successful talent management 4. Examples of successful talent management 5. Discuss time management.	(i) Description of communication and motivation in talent management (ii) Lecture on the model of talent management. (iii) Lecture on successful talent management. (iv) Provide examples of successful talented students. (v) Lecture on time management (vi) Review of the day (vii) Provision of homework that aims to identify challenges other than those mentioned in the session
**Third session** 6 Discussion of the talent management Process	1. Complete the discussion on talent management. 2. Challenges and Considerations 3. Identify the process of talent management.	(i) Sharing homework from the previous session (ii) Brainstorming about talent management main components, including experience, transformation, process, and knowledge (iii) Group discussion about the process of talent management
**Fourth session** (personal talent management plan)	1. Create a personal talent management plan. 2. Clarify the next intervention.	(i) Participants individually talent management learning and reflection (ii) Discuss talent management learning and experience (iii) Discussion about talent management learning and social interaction (iv) Concluding all sessions

#### Phase three: evaluation talent management educational intervention phase

After the implementation phase of the educational intervention, the participating students were reassessed for their understanding of talent management, satisfaction, self-efficacy, and student achievement effectiveness using the same tools and comparing their results with the pre-test.

### Control group

Nursing students in the control group received only the content from the first and second sessions. They don’t participate in talent management activities or develop a personal talent management plan. Unlike the study group, the control group did not participate in the reflection session or the three-week talent management intervention.

### Statistical approach

All statistical analyses were performed using SPSS for Windows version 2.0 (SPSS, Chicago, IL). The normality of data was first tested with a one-sample Kolmogorov-Smirnov test. Continuous data were normally distributed and were expressed in mean standard deviation (SD). Categorical data were expressed in numbers and percentages. The Chi-square (or Fisher’s exact test when applicable) test was used for comparison of variables with categorical data.

Fisher’s exact test can only be used with categorical data, and categorical data cannot have abnormal data distribution since it is not displayed by mean and standard deviation, and More than 20% of cells are less than 5, For a 2 × 2 used, Fisher’s exact test.

The two groups were compared with an independent t-test, while paired groups (pre- and post-intervention) were compared by a paired t-test. A correlation coefficient test was used to test for correlations between two variables with continuous data. The reliability (internal consistency) test for the questionnaires used in the study was calculated. The threshold of significance is fixed at a 5% level (p-value). The results were considered significant when the probability value was less than 5% (*p* ≤ 0.05). The smaller the p-value obtained, the more significant the result.

### Ethics clearance and approval for participation

The ethical approval to conduct this study was obtained from the standing committee of scientific research ethics at Shaqra University, Saudi Arabia (Reference number: ERC_SU_S_202300013). The questionnaire included a statement related to the aim and nature of the study. All participants who chose the word agree to give their informed consent before beginning their response to the sheet. The respondents were guaranteed the privacy and confidentiality of their answers, the voluntary nature of their involvement, and the fact that their absence would not hurt their grades or result in any negative outcomes. This ensures that participants can freely choose to participate without the fear of academic repercussions. Participants have given their informed consent under the criteria outlined in the Helsinki Declaration. It was determined that participants had the right to withdraw from the study at any time. The right to withdraw was communicated both verbally and in writing at the outset of the study. The authors confirm that all methods were performed according to the relevant guidelines and regulations.

## Results

Table 2: The variables of the study sample were homogenous in the two groups (study and control). It’s clear in Table (1) that the mean age of the nursing students was 21.3 years (± 2.5), and the majority of nursing students (82.8% and 75.3% among the study and control groups, respectively) were in the age group between 20 and less than 23 years, also were single (97.8% and 93.5% among study and control groups, correspondingly), majority of both groups were female and 97.8% and 94.6% of study and control groups, respectively, joined nursing department based on their desires. Furthermore, 90.3% and 92.5% of the study and control groups, respectively, did not work alongside studying.

**Table 2:** Demographic characteristics of studied nursing students (*N* = 186)

	Study (*n* = 93)		Control (*n* = 93)		Chi-Square / Fisher’s exact test	
	*N*	%	*n*	%	Test of significance	*P*
Age (Years)						
17 – <20	9	9.7	12	12.9		
20 – <23	77	82.8	70	75.3		
23–25	7	7.5	11	11.8	1.650	0.438^$^
Mean ± SD	21.3 ± 2.5		20.9 ± 3.1		t = 0.968^#^	0.334^#^
Gender						
Female	49	52.7	48	51.6		
Male	44	47.3	43	46.2		
Level of education						
3rd	47	50.0	47	50.0		
5th	34	32.0	34	32.0		
7th	12	18.0	12	18.0	0.209	0.646^$^
Marital status						
Single	91	97.8	87	93.5		
Married	2	2.2	6	6.5	2.089	0.148^@^
Working alongside studying						
No	84	90.3	86	92.5		
Yes	9	9.7	7	7.5	0.273	0.601^$^
Joining the nursing department based on a personal desire						
No	2	2.2	5	5.4		
Yes	91	97.8	88	94.6	1.335	0.247^@^

Note: @: Fisher’s exact test, #: independent t – test, $: Chi – square test

P Significance * Significant (*p* ≤ 0.05)

Highly statistically significant at ** *p* ≤ 0.001

**Fig. 1:**
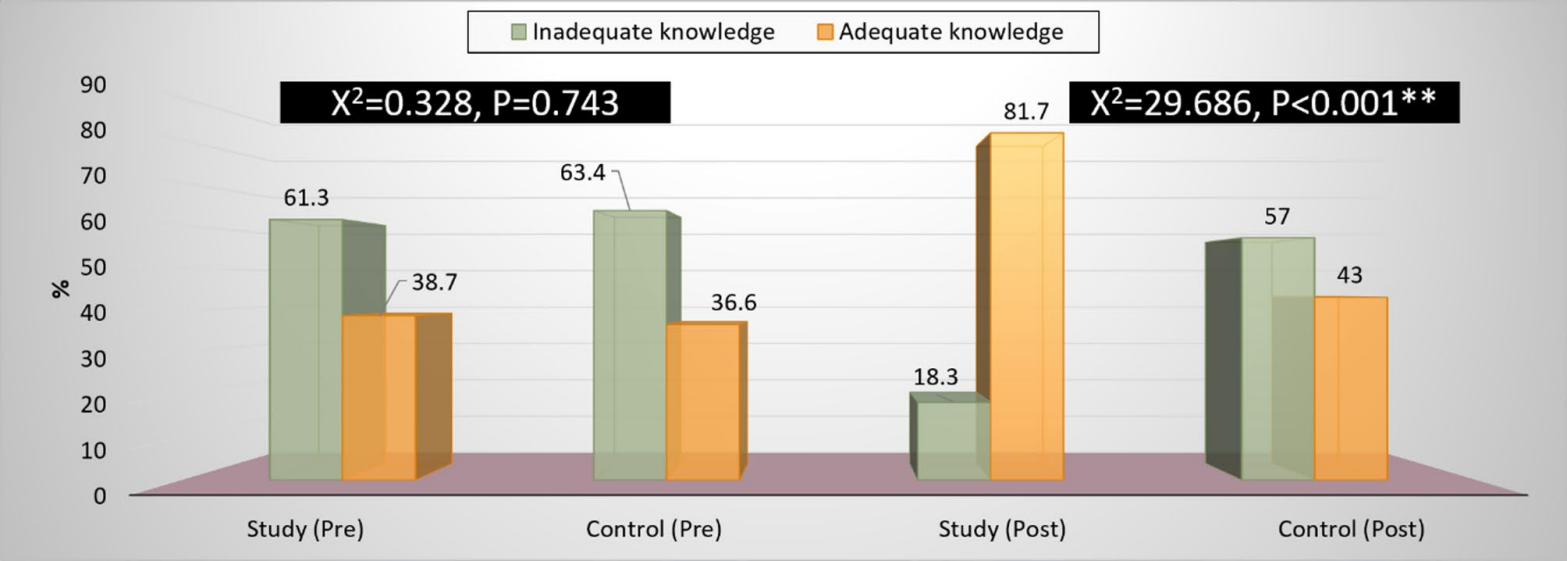
Comparison of total knowledge level at pre and post-intervention. X2: Chi-square test

Figure 1 Comparison of knowledge score and level at pre- and post-talent management educational intervention programs. As shown, the low total mean for knowledge was (38.7, 36.6) for both study and control groups correspondingly at pre-educational intervention. Moreover, the table revealed that the highest mean score regarding knowledge (81.7, 43.0) for the study and control group respectively intervention program with a highly significant improvement (*P* < 0.0001).

Table 3 Comparison of talent management competency and total scores at pre- and post-talent management educational intervention programs. As shown, the low total means for talent management (104.8 and 103.4) for both the study and control groups, respectively at the time of the pre-talent management educational intervention. Moreover, the table revealed the highest mean score regarding talent management dimensions (talent attraction (39.4), talent development (38.5), and talent retention (42.5) for the study group post-intervention program with a highly significant improvement (*P* ≤ 0.0001).

**Table 3:** Comparison of talent management competency and total scores at pre- and post-talent management educational intervention programs (*N* = 186)

Talent management domains	Study				Control				P3 value
	Mean	SD	Min – Max	P1 value	Mean	SD	Min – Max	P2 value	
Talent attraction									
Pre	35.0	11.6	12–58	t = 2.821, *P* = 0.005*	34.7	10.1	15–55	t = 0.644, *P* = 0.519	t = 2.815, *P* = 0.005*
Post	39.4	9.5	20–58		35.6	8.9	18–53		
Talent development									
Pre	34.5	11.2	12–57	t = 2.521, *P* = 0.013*	33.9	10.8	12–56	t = 0.793, *P* = 0.428	t = 2.318, *P* = 0.022*
Post	38.5	10.2	18–59		35.1	9.8	16–55		
Talent retention									
Pre	35.2	12.1	11–59	t = 4.340, *P* < 0.001**	34.8	10.9	13–57	t = 1.154, *P* = 0.249	t = 4.097, *P* < 0.001**
Post	42.5	10.8	21–64		36.5	9.1	18–55		
Total Talent Management score									
Pre	104.8	32.8	39–170	t = 3.389, *P* < 0.001**	103.4	31.1	41–166	t = 0.841, *P* = 0.401	t = 2.980, *P* = 0.003*
Post	120.4	29.9	61–180		107.2	30.5	46–168		

Note:

a paired t-test (P1) = Comparison of each dimension of talent management among study groups pre- and post-intervention

Paired t-test (P2) = Comparison of each dimension of talent management among the control group pre- and post-intervention

Independent t-test. (P3) = Comparison of each dimension of the talent management study and the control group’s post-intervention

P Significance * Significant (*p* ≤ 0.05)

Highly statistically significant at ** *p* ≤ 0.001

Table 4 demonstrates a comparison of students’ satisfaction with pre- and post-talent management educational intervention programs of students’ satisfaction dimensions and total scores among studied nursing students, pre- and post-talent management educational intervention for study and control groups. As shown, the low total mean of satisfaction was (92.5 and 92.3) for both the study and control groups, respectively, at pre-talent management educational intervention. Additionally, the post-intervention program for the study and control group revealed the highest mean scores regarding students’ satisfaction dimensions (satisfaction with faculty staff members, support staff, and employees (76.7), satisfaction with the physical environment (33.5), and satisfaction with the learning courses used in the faculty (38.1) for the study group post-intervention program with a highly significant improvement at (*P* ≤ 0.0001). Also, the result confirmed that a high level of satisfaction was 82.8% after the talent management educational intervention of the study group with a highly significant improvement (*P* ≤ 0.0001). (Supplementary Fig).

**Table 4:** Comparison of students’ satisfaction at pre- and post- talent management educational intervention programs (*N* = 186)

Students’ satisfaction questionnaire	Study				Control				P3 value
	Mean	SD	Min – Max	P1 value	Mean	SD	Min – Max	P2 value	
Satisfaction with faculty staff members, support staff, and employees									
Pre	47.9	8.9	30–66	t = 18.663, *P* < 0.001**	46.8	9.1	29–65	t = 0.917, *P* = 0.360	t = 17.597, *P* < 0.001**
Post	76.7	11.9	53–101		48.1	10.2	28–69		
Satisfaction with the physical environment									
Pre	21.5	10.5	1–43	t = 10.148, *P* < 0.001**	21.9	9.8	2–42	t = 1.047, *P* = 0.296	t = 14.461, *P* < 0.001**
Post	33.5	4.7	24–43		23.1	5.1	13–33		
Satisfaction with the learning courses used in the faculty									
Pre	23.1	9.1	5–41	t = 13.775, *P* < 0.001**	23.6	8.6	6–41	t = 1.461, *P* = 0.145	t = 17.368, *P* < 0.001**
Post	38.1	5.3	28–49		25.1	4.9	15–35		
Total Satisfaction level									
Pre	92.5	15.8	61–124	t = 26.091, *P* < 0.001**	92.3	15.1	62–123	t = 1.942, *P* = 0.053	t = 27.117, *P* < 0.001**
Post	148.4	13.3	122–175		96.3	12.9	71–122		

Note: Paired t-test (P1) = Comparison of the study group’s satisfaction levels before and after the intervention for each dimension

Paired t-test (P2) = Comparison of the control group’s level of satisfaction with each dimension before and after the intervention

Independent t-test (P3) = A comparison between the control groups and every aspect of the students’ satisfaction study after the intervention

P Significance * Significant (*p* ≤ 0.05)

Highly statistically significant at ** *p* ≤ 0.001

Table 5 reveals that mean scores were increased in total students’ self-efficacy, ranging from 13.9 ± 2.0 pre-intervention to 19.1 ± 1.7 post-intervention for the study group with a highly significant difference at (*p* ≤ 0.0001). The control group had the lowest total mean of self-efficacy (15.2 ± 2.9) post-intervention.

**Table 5:** Comparison of students’ Self-efficacy at pre and post talent management educational intervention programs (*N* = 186)

Students’ Self-efficacy questionnaire	Pre-intervention						Post-intervention					
	Study		Control		Chi-Square		Study		Control		Chi-Square	
	*n*	%	*n*	%	X^2^	*P*	*n*	%	*n*	%	X^2^	*P*
I expect to do as well as or better than other students in my subjects												
Never	40	43.0	42	45.2			3	3.2	39	41.9		
Rarely	13	14.0	10	10.8			4	4.3	12	12.9		
Sometimes	22	23.7	20	21.5			21	22.6	23	24.7		
Always	18	19.4	21	22.6	0.766	0.857	65	69.9	19	20.4	60.138	< 0.001**
I am confident that I will do my best in the college laboratories												
Never	27	29.0	29	31.2			6	6.5	25	26.9		
Rarely	12	12.9	13	14.0			9	9.7	17	18.3		
Sometimes	29	31.2	28	30.1			20	21.5	32	34.4		
Always	25	26.9	23	24.7	0.212	0.975	58	62.4	19	20.4	36.629	< 0.001**
I believe that I can apply the information and skills in the academic subjects												
Never	36	38.7	35	37.6			5	5.4	32	34.4		
Rarely	11	11.8	15	16.1			4	4.3	19	20.4		
Sometimes	21	22.6	23	24.7			23	24.7	25	26.9		
Always	25	26.9	20	21.5	1.275	0.734	61	65.6	20	21.5	50.286	< 0.001**
I am confident that I will do well in the exams												
Never	34	36.6	32	34.4			5	5.4	29	31.2		
Rarely	13	14.0	11	11.8			5	5.4	16	17.2		
Sometimes	20	21.5	23	24.7			21	22.6	26	28.0		
Always	26	28.0	27	29.0	0.455	0.928	62	66.7	22	23.7	42.282	< 0.001**
I think I can get an excellent grade in all subjects												
Never	35	37.6	37	39.8			7	7.5	31	33.3		
Rarely	14	15.1	16	17.2			3	3.2	21	22.6		
Sometimes	21	22.6	22	23.7			23	24.7	26	28.0		
Always	23	24.7	18	19.4	0.821	0.844	60	64.5	15	16.1	55.841	< 0.001**
Total students’ self-efficacy Mean ± SD	**13.9 ± 2.0**		**14.1 ± 2.3**		**t = 0.632**	**0.527**	**19.1 ± 1.7**		**15.2 ± 2.9**		**t = 11.188**	< 0.001**

Independent t–test - X2: Chi-square test

**Fig. 2:**
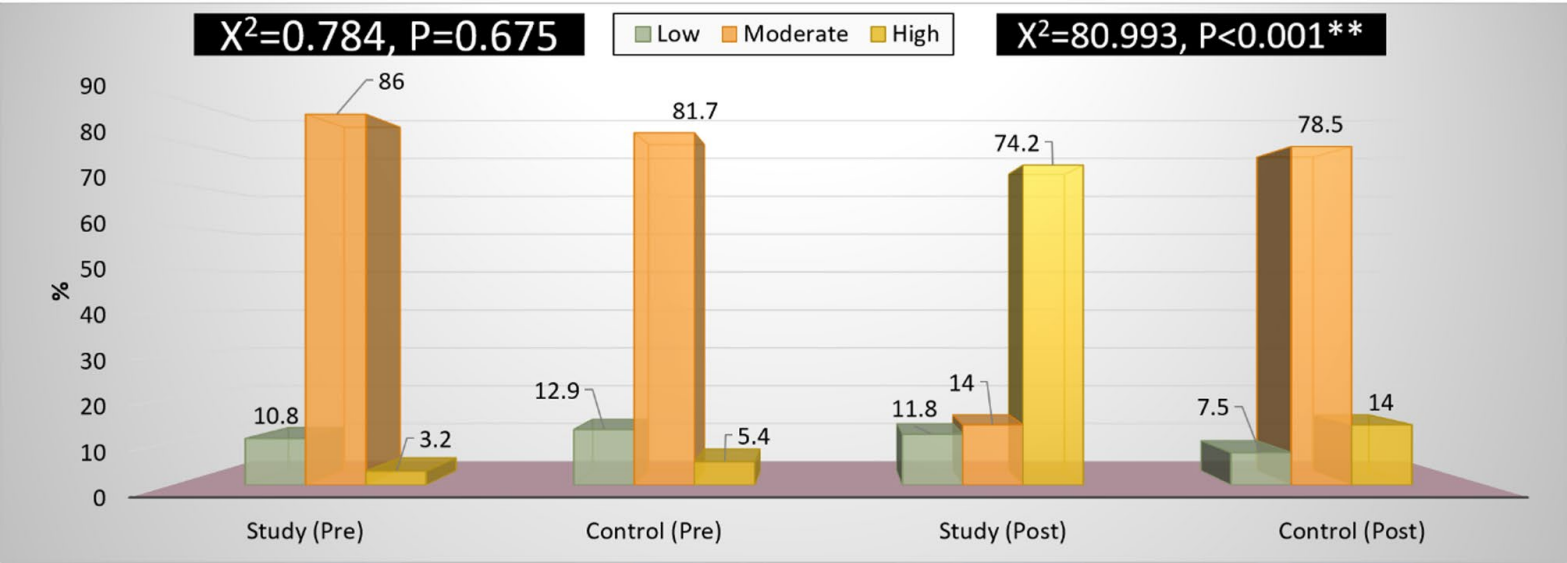
Comparison of Students’ academic achievement questionnaire total at pre and post-intervention. *X2: Chi square test*

Figure 2 The result reported that the nursing students only 3.2% and 5.4% of the studied students in both the study and control groups had a high level of academic achievement with the pre-talent management educational intervention. In the study group, talent management post-educational intervention resulted in a highly significant improvement (*P* < 0.0001) and a high level of academic achievement of 74.2%.

Table 6 shows the correlation between knowledge, talent management, satisfaction, self-efficacy, and academic achievement among the studied nursing students after intervention. It was noticed that there was a significantly positive correlation between students’ Knowledge, talent management competency level, satisfaction, self-efficacy, and academic achievement (*P* ≤ 0.0001).

**Table 6:** Correlation between students’ knowledge, talent management, students’ satisfaction, students’ Self-efficacy, and students’ academic achievement of the study group after application of talent management educational intervention programs

	Knowledge		Talent management		Students’ satisfaction		Students’ Self-efficacy		Students’ academic achievement questionnaire	
	*R*	*p*	*r*	*P*	*r*	*p*	*r*	*p*	*r*	*p*
Knowledge			0.434	< 0.001**	0.219	0.035*	0.805	< 0.001**	0.395	< 0.001**
Talent management	0.434	< 0.001**			0.352	< 0.001**	0.369	< 0.001**	0.218	0.036*
Students’ satisfaction	0.219	0.035*	0.352	< 0.001**			0.221	0.033*	0.257	0.013*
Students’ Self-efficacy	0.805	< 0.001**	0.369	< 0.001**	0.221	0.033*			0.385	< 0.001**
Students’ academic achievement questionnaire	0.395	< 0.001**	0.218	0.036*	0.257	0.013*	0.385	< 0.001**		

Note

r: for Pearson correlation

P Significance * Significant (*p* ≤ 0.05)

Highly statistically significant at ** *p* ≤ 0.001

Table 7 The results of the binary multiple logistic regression analysis indicate a significant influence of talent management on various study variables within the study group. **Knowledge**: The coefficient (B = 0.408) suggests that effective talent management is associated with an increase in knowledge, with a statistically significant p-value (< 0.001). The odds ratio (Exp(B) = 1.503) indicates that for every unit increase in talent management, the odds of increased knowledge are approximately 1.5 times higher, with a 95% confidence interval (CI) ranging from 1.179 to 1.916. **Students’ Satisfaction**: The analysis shows a positive relationship between talent management and students’ satisfaction (B = 0.105, *p* = 0.003). The odds ratio (Exp(B) = 1.111) indicates a 11.1% increase in the odds of satisfaction with each unit increase in talent management, supported by a CI of 1.037 to 1.189. **Students’ Self-efficacy**: A strong positive correlation is observed (B = 0.716, *p* < 0.001), suggesting that talent management significantly enhances students’ self-efficacy. With an odds ratio of 2.046, talent management doubles the odds of improved self-efficacy, with a CI of 1.462 to 2.864. **Students’ Academic Achievement**: Finally, talent management is also positively associated with students’ academic achievement (B = 0.078, *p* < 0.001). The odds ratio (Exp(B) = 1.081) indicates a modest increase of 8.1% in the odds of academic success per unit increase in talent management, with a CI of 1.035 to 1.130.

Overall, these findings underscore the critical role of talent management in fostering knowledge, satisfaction, self-efficacy, and academic achievement among students, highlighting its importance in educational settings.

**Table 7:** The results of binary multiple logistic regression analysis to study the influence of talent management on different study variables for the study group

	B	SE	Sig. (*P* value)	EX (B)	Confidence interval (CI)	
					Lower limit	Upper limit
The influence of talent management on knowledge	0.408	0.124	< 0.001**	1.503	1.179	1.916
The influence of talent management on Students’ satisfaction	0.105	0.035	0.003*	1.111	1.037	1.189
The influence of talent management on Students’ Self-efficacy	0.716	0.172	< 0.001**	2.046	1.462	2.864
The influence of talent management on Students’ academic achievement questionnaire	0.078	0.023	< 0.001**	1.081	1.035	1.130

*Significant (*P* < 0.05)

B = Logistic Regression Coefficient

SE = Standard Error of B

P = Significance level

Exp (B) = Estimated Odds Ratio

## Discussion

Identifying talent and helping individuals reach their maximum potential are two of the main goals of human resource management on a global scale. These are goals that both developed and developing economies and cultures work to accomplish, mostly with the help of universities and the educational system. Our university’s primary goal is to identify gifted individuals and assist them in developing their abilities. This is becoming a more common undertaking across all academic programs and levels of education, not only because it is a wonderful chore and endeavor for the university but also because it is in line with society’s expectations.

Focused educational interventions can significantly improve the academic performance, self-efficacy, and satisfaction of future nurses. By incorporating talent management strategies into nursing education, institutions can foster an environment that promotes both professional and personal growth. These interventions, which include skill development, cooperative learning opportunities, and mentoring programs, make nursing students feel more confident and content with their professions in general. Research indicates that when students feel supported and equipped with the necessary abilities, they do better academically. This leads to a more competent and satisfied nursing workforce that is ready to meet the needs of healthcare delivery [44].

The purpose of the current study was to enhance self-efficacy, satisfaction, and academic achievement among future nurses through talent management educational intervention. The results of the current study showed that nursing students’ general knowledge of their talent management and management techniques improved after the implementation of the educational intervention, with a highly significant improvement (*P* < 0.0001) in the study group. This outcome might have to do with how well the talent management program has worked to broaden nursing students’ understanding. The substantial improvement (*P* < 0.0001) indicates that the intervention not only kept students interested but also gave them the knowledge and abilities they needed for their line of work. Participants’ confidence and competence are expected to increase as a result of these programs’ promotion of a more thorough understanding of nursing principles. This emphasizes the value of focused teaching methods in healthcare education and professional development. This result agreements with [45] the implementation of talent management of nursing in hospital which was conducted at Damanhur National Medical Institute to examine the Effect of Talent Management Training Program on Head Nurses Leadership Effectiveness and who revealed that a highly statistically significant improvement in head nurses’ knowledge and practice of talent management was found in the post-intervention phase of the study group, Also, this results in congruence with [46] Study was conducted at Benha University Hospital, Egypt to evaluate the effect of Educational Program about Talent Management and Organizational Excellence and who revealed that there was highly statistical significant improvement in the nursing managers’ levels of talent management knowledge and activities, and organizational excellence after intervention the program. Moreover [47], who examines how talent management procedures affect knowledge creation, indicated that the main finding of this study is that talent management (TA, TD, and TR) has a highly significant and considerable impact on knowledge creation in Australia.

As yielded by the current study, showed the highest mean score regarding knowledge for the study and control group, respectively intervention program with a highly significant improvement (*P* < 0.0001). while minor nursing students’ improvement in the control group. this may be related to students hearing about talent management from other colleagues in the faculty, or it may be related to students gaining clear and accurate information regarding academic regulations and requirements through academic advising. This result is supported by [48]. We examine the impact of academic advising on student performance and demonstrate that there is a noteworthy correlation between academic advising and student achievement. A variety of demands related to academic advising have been identified, and students exhibit a preference for advisors who are reliable and supportive.

The result was incongruent with [49], who stated that while some studies advocate for the integration of leadership and management skills into nursing education, they also point out that many nursing programs still prioritize clinical skills over management competencies. This gap can lead to students feeling unprepared for the leadership aspects of their roles, suggesting that improvements in awareness may not always translate into effective application in practice. Also, in contrast with these findings, a study was done by [50] who look into the effects of talent management practices in higher education and discovered that most of these institutions’ current talent management strategies are largely ineffective because they don’t guarantee talent retention, inspire, or engage the institution’s talent in addition to improving achievement.

The results of the current study regarding talent management competency levels the results showed that nursing students’ employment of competitive strategies increased statistically significantly with the deployment of post-training strategies. This study suggested that, as a result of the program’s execution, nursing students became more conscious of and adept at using their influence. They also mastered acting with more assurance and confidence. This interpretation is supported by [51] showed that despite major implementation issues, participants in a talent management program unanimously acknowledged the strategic significance of the program, and an effective talent management program with statistically significant improvement. Similarly, the findings of [52]. They stated that training is the most effective technique to broaden knowledge and change nursing managers attitudes, values, and views. The study discovered that all talent management domains showed highly statistically significant differences and favorable associations (*p* < 0.01**), with post-intervention talent management skill levels being high compared to poor pre-intervention levels. Additionally [53], ran a training course for talent management strategies of private higher education institutions in Botswana, and they reported that in stated that there is still work to be done because managers in these institutions lack the knowledge and skills necessary to plan and carry out talent management programs.

With regard to students’ satisfaction, the current study revealed a highly statistically significant rise in the usage of post-talent management educational intervention programs of students’ satisfaction dimensions and total scores among the studied nursing students. This outcome might have to do with how well educational interventions in talent management raise the motivation and engagement of nursing students. These initiatives probably provide a more positive learning environment by addressing several aspects of student satisfaction, like academic assistance, mentorship, and skill development. As a result, the high satisfaction ratings demonstrate both the caliber of the educational process and the effective matching of program goals with students’ needs and goals, which eventually helps them succeed academically and prepare for the workforce. All of this increased the students’ satisfaction. This finding supports the claims made by [54], who argued that talented students expressed high levels of satisfaction with the administration and professors; they expressed only moderate satisfaction with the facilities and equipment, student relationships, enrichment programs, and teaching techniques. Additionally [55], who looked into how talent management practices affected employee satisfaction in universities and higher education institutions claimed that these practices had a statistically significant impact on employee satisfaction among professors, teachers, and other staff members. Moreover [56], Research shows that satisfaction with nursing education programs and job satisfaction one year after graduation are significantly correlated. This suggests that the foundations laid during nursing school, particularly talent management strategies, will have a significant impact on future job satisfaction. Also [57], Research indicates that nursing students who take talent management courses are more satisfied with their jobs. Numerous factors, such as the type of training and assistance they received, influence this happiness. There is a statistical correlation between nursing students’ satisfaction and talent management techniques.

Concerning the students’ self-efficacy, this result reveals that mean scores were increased in total students’ self-efficacy post-intervention for the study group with a highly significant difference at (*p* ≤ 0.0001). Effective personnel management techniques may have contributed to this outcome by offering opportunities for growth and individualized support. Students probably felt more confident in their skills and had a higher sense of self-efficacy as a result of recognizing and fostering their unique strengths. Furthermore, students may have been encouraged to establish and accomplish more ambitious goals by the structured feedback and mentoring provided by talent management, which strengthened their confidence in their ability to succeed. The findings of this study are consistent with those of [58], who showed that talent management has a highly substantial positive impact on the personnel under study’s sense of self-efficacy. Moreover [59], added that assisting pupils in raising their degree of self-efficacy results, students can learn more effectively, being more upbeat, and concentrating on improving their academic achievement, all of which help to keep them out of trouble with the law. Moreover [60], argued that the talent management self-efficacy score of nursing students significantly increased after sessions. while, this result is in contrast with [61], they found that the reason talent management is probably insufficient to influence students’ self-efficacy is unclear.

Regarding nursing students’ academic achievement, the current study found that the overall means of academic achievement at the post-intervention program for the study group had a highly significant improvement (*P* ≤ 0.0001) in the highest mean score regarding students’ academic achievement dimensions (academic achievement, extracurricular activities, student interaction, student behavior, and student attendance). This outcome might be linked to the application of successful talent management techniques that raise students’ motivation and engagement levels and enable them to make greater use of their interests and strengths. Schools can design specialized learning experiences that foster deeper comprehension and skill development, which will ultimately increase academic performance by recognizing and fostering particular abilities. Furthermore, these tactics might create a nurturing classroom atmosphere that promotes cooperation and individual development. This study was supported by [62]. A study on private higher education institutions (PHEIs) found that a conducive academic environment and the systematic use of knowledge and talent management improve organizational performance. This implies that effective TM strategies can lead to better academic achievement and institutional success. Also [63], who made the report that the long-term competitive advantage of higher education institutions (HEIs) is significantly increased by integrated talent management (TM) strategies, which include talent attraction, development, and retention. This is particularly crucial as schools compete for the same talent pool, which is considered an essential resource.

In support of this finding, a study by [64] that looked into the best practices for talent management and organizational success reaffirmed the need for talent management for businesses looking to grow and succeed in a sustainable way because it retains top talent and high-potential students while increasing productivity. In conclusion, a methodical and comprehensive approach to people management can provide a measurable advantage and ensure long-term survival and success. Additionally [65], looked studied how teachers felt about their students’ academic success and found that teachers’ professional competence, mentoring, care, intellectual interest, and role modeling all affect students’ academic success. Talent management techniques are the source of these consequences. Students’ academic performance is also influenced by their educational attainment.

As regards the correlation between knowledge, talent management competency level, satisfaction, self-efficacy, and academic achievement among the studied nursing students after intervention. It was noticed that there was a significantly positive correlation between students’ knowledge, talent management competency level, satisfaction, self-efficacy, and academic achievement (*P* ≤ 0.0001). This outcome might be linked to the talent management program’s all-encompassing strategy, which probably improved nursing students’ knowledge and abilities and raised their sense of self-efficacy. Higher academic achievement may have resulted from students’ increased overall contentment with the learning environment as they grew more confident in their skills. The relationships among these elements imply that good talent management not only promotes personal development but also builds a positive school climate, both of which have a big influence on student results. The correlation between knowledge, talent management competency level, satisfaction, self-efficacy, and academic achievement among nursing students is a critical area of research, particularly following interventions aimed at enhancing these factors. Improvements in knowledge and talent management have been found to directly correlate to improved levels of satisfaction and self-efficacy, which in turn boost academic accomplishment. These characteristics are significantly positively correlated in recent studies. For instance, a study conducted on nursing students found that academic self-efficacy is crucial for improving academic performance and managing stress. According to the results, indicating a favorable correlation between the characteristics mentioned, pupils who have a higher sense of self-efficacy are likely to perform better academically [66].

This result is consistent with those of [67], who documented that there was a highly statistically significant positive correlation between the overall score of talent management, self-efficacy, and organizational effectiveness among staff nurses. this result is also in agreement with [68], who found that the relevance of self-efficacy and academic achievement will enable prompt interventions, fostering success and averting failure and dropout in higher education. Also [69], observed that in both groups, achievement was significantly positively related to academic self-efficacy, and Life satisfaction was positively related to academic satisfaction was related to critical thinking and academic self-efficacy. Furthermore [70], presented findings indicating that students’ accomplishments acted as a mediator in the association between academic self-efficacy and achievement/satisfaction. On the other hand, this finding contradicts that of [71], who found that although self-efficacy is substantial, it does not always correspond with academic accomplishment, especially in high-stress contexts like clinical settings, where outcomes can be greatly influenced by outside influences.

Finally, organizations are reevaluating how they hire, train, retain, and use their workforces since talent management has become such a critical practice to economic growth and corporate success. Health care organizations must gamble on people rather than on factories, technologies, or even gear or equipment because they cannot create money without the ideas, labor, talent, and abilities of knowledge workers. Talent management strategies are critical to organizational performance and long-term growth because they assist organizations in retaining top talent and high-potential workers while increasing productivity [64], also, total work innovation and total performance [72].

## Conclusion and recommendations

The study confirms the positive impact of a talent management educational intervention on nursing students’ knowledge, competencies, satisfaction, self-efficacy, and academic performance. Post-intervention, students with a strong understanding of talent management exhibited greater satisfaction and self-efficacy, leading to enhanced academic achievement. These findings emphasize the need to integrate talent management strategies into nursing education to develop students’ professional competencies and career readiness. Including educational programs on talent management competencies in their upcoming training to support the healthcare services’ marketability, profitability, and viability. Designing nursing curricula that encourage students’ autonomy and critical thinking skills that are required for their academic satisfaction, academic self-efficacy, and achievement. Providing continuous, adequate and constructive feedback to students about their academic performance is needed for academic achievement.

## Limitations of the study

There are several advantages and disadvantages to the study on improving nursing students’ academic performance, self-efficacy, and satisfaction using a talent management educational intervention. The balanced sample size of 186 students, split equally between the research and control groups, is a noteworthy strength that minimizes bias and enables efficient comparison. Additionally, the findings are more broadly applicable to the larger nursing student population due to the proportionate representation of participants across program levels (2nd, 3rd, and 4th year). Limitations, however, include the possibility that outside variables, such as different degrees of prior knowledge or motivation, could have an impact on student performance and were not taken into account during the study’s design. Additionally, the validity of the results may be impacted by subjective bias introduced by the use of self-reported measures to gauge self-efficacy and satisfaction. Overall, even though the study offers insightful information, these limitations point to the necessity of additional research to validate and build upon its conclusions.

Undergraduate nursing students in Saudi Arabian state universities usually do not have access to a specialized training program that focuses only on talent management. Patient care, clinical skills, and basic nursing knowledge are the main focuses of their program. There is typically no specific program designed to build talent management skills within the nursing school framework, even though elements of talent management may be incorporated into more general coursework.

## Directions for future research

Future research should focus on longitudinal studies to evaluate the long-term impact of talent management interventions on career progression and professional development in nursing. Expanding the study to multiple institutions will help assess the sustainability of knowledge retention and competency development over time. Additionally, further research should explore factors influencing academic satisfaction, self-efficacy, and achievement, as well as the role of organizational culture, faculty engagement, and institutional policies in enhancing the effectiveness of talent management in nursing education. Gathering participant feedback on the practicality and effectiveness of these strategies will also provide valuable insights for future improvements.

## Electronic supplementary material

Below is the link to the electronic supplementary material.

Supplementary Material 1

Supplementary Material 2

Supplementary Material 3

## Data Availability

The data that support the findings of this study are available from the corresponding author upon reasonable request. All data generated or analyzed during this research are included in this manuscript. The authors confirm that all methods were performed according to the relevant guidelines and regulations.
