# Supplementary material for: Empowering future nurses: enhancing self-efficacy, satisfaction, and academic achievement through talent management educational intervention
Source: BMC Nurs. 2025 Jul 7;24:875. doi: 10.1186/s12912-025-03512-z (PMC12235881; doi:10.1186/s12912-025-03512-z)
Supplement: Supplementary file 1 — Supplementary Material 1 [file 12912_2025_3512_MOESM1_ESM.pdf]

talent management knowledge questionnaire

Thanks for your participation in this program and please fill the questionnaire and be sure that all information will be kept confidential.

1-Demographic data

* **G**ender :(male – female)

* Your name:

*Level of education

*Marital status:

*Age

2- Talent management knowledge questionnaires

I-True and False questions

-Read the following and beside each statement put either (T) if the statement is true or (F) if False:

No	***Items***	
1	The talent management cannot evaluate clinical practice.	
2	Talent management is a way of managing successful human resources.	
3	The talent management improves the communication between the nurse's students.	
4	Talent management enables nurses to gain confidence and good practice.	
5	During Talent management potential problems can be discussed between nurses.	
6	Areas of work that need improvement can be identified at any stage by Talent Management.	
7	Training and development needs can be identified by Talent Management	
8	Talent management increases motivation and job satisfaction	
9	Talent Management is beneficial for professional development for nurses.	
10	Talent Management is beneficial for improves the quality of patient care.	
11	Talent Management isn't considered to be best clinical practice for all student's.	
12	Talent Management should be safe and supportive.	

13	Lack of continual and ongoing leadership is one of the barriers to good Talent Management.	
14	Talent Management develops the nurses' skills and knowledge through training.	
15	Talent Management decreases the feelings of isolation and distress for students.	
16	Quality of Talent Management must be aware of the point's strengths and weaknesses.	
17	Talent Management should be good communicator	
18	Increasing nursing' retention rates is one of the benefits of talent management.	
19	Talent retention is a step to convince head nurses cannot stay with the organization as long as possible	
20	Talent development is the investment of knowledge, skills, and human capital capacities that unlock the performance potential of critical tasks.	

II-Multiple choice

-Circle around one best given answer for each question

1-All of the following are Success Factors of Talent Management except

a) Leadership commitment.
b) Performance management.
c) Organizational culture.
d) Attraction and selection.

2-The Talent management categories is;

a) Attraction and selection.
b) Retention.
c) Development.
d) All of the above.

3- All of the following are Talent management theories except

a) psychological contract.
b) Organizational Support Theory.
c) Organizational Commitment Theory.

d) Leadership Talent Mindset.

4- One of the elements of the Talent management practices is;

a) Organizational Talent Brand..

b) Talent Onboarding and Deployment

c) Talent Performance Management and Recognition.

d) All of the above

5- All of the following are barriers of talent management except

a) lack of strong talent pool.

b) inadequate time .

c) lack of performance management standards.

d) Organizational Commitment.

6-Role of faculty in talent management is:

a. Teacher
b. Innovator and leader.
c. Helper.
d. All of the above.

7-The process of talent management includes:

a) Planning phases.
b) Attracting phases.
c) Selecting phases.
d) All of the above.
