# Supplementary figures and images for: Empowering future nurses: enhancing self-efficacy, satisfaction, and academic achievement through talent management educational intervention
Source: BMC Nurs. 2025 Jul 7;24:875. doi: 10.1186/s12912-025-03512-z (PMC12235881; doi:10.1186/s12912-025-03512-z)

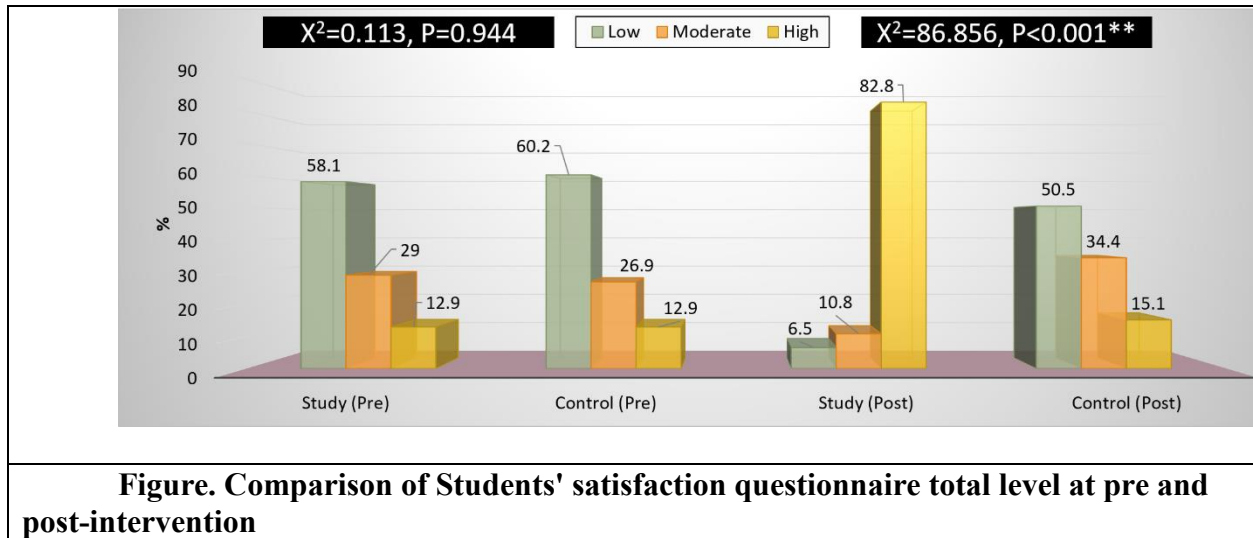

Supplement: Supplementary file 2 — Supplementary Material 2 [file 12912_2025_3512_MOESM2_ESM.pdf]
