# Supplementary material for: Empowering future nurses: enhancing self-efficacy, satisfaction, and academic achievement through talent management educational intervention
Source: BMC Nurs. 2025 Jul 7;24:875. doi: 10.1186/s12912-025-03512-z (PMC12235881; doi:10.1186/s12912-025-03512-z)
Supplement: Supplementary file 3 — Supplementary Material 3 [file 12912_2025_3512_MOESM3_ESM.pdf]

Table. Comparison of knowledge score and level at pre- and post-talent management educational intervention programs (N=186)

	Pre-intervention						Post-intervention					
	Study		Control		Test of significance	P value	Study		Control		Test of significance	P value
	n	%	n	%			n	%	n	%		
Knowledge level												
Inadequate knowledge	57	61.3	59	63.4			17	18.3	53	57.0		
Adequate knowledge	36	38.7	34	36.6	X^{2}=0.091	0.762	76	81.7	40	43.0	X^{2}= 29.686	<0.001**
Mean ±SD	13.6 ±8.7		13.2 ±7.9		t =0.328	0.743	21.5 ±6.9		14.2 ±7.3		t =7.008	<0.001**

Table. Comparison of Students' academic achievement dimensions pre-and post- talent management educational intervention programs (N=186)									
**Students' academic achievement**	**Study**				**Control**				
	**Mean**	**SD**	**Min – Max**	**P1 value**	**Mean**	**SD**	**Min – Max**	**P2 value**	**P3 value**
**Academic performance**									
**Pre**	18.2	8.7	1 – 36	T=5.573, P<0.001**	18.6	9.3	0 – 37	T=0.884, P=0.377	T=4.452, P<0.001**
**Post**	26.6	11.5	4 – 50		19.8	9.2	1 – 38		
**Extracurricular activities**									
**Pre**	18.0	8.6	1 – 35	T=5.830, P<0.001**	18.2	9.0	0 – 36	T=0.979, P=0.328	T=4.570, P<0.001**
**Post**	25.5	8.8	8 – 43		19.5	9.1	1 – 38		
**Student's interaction**									
**Pre**	19.6	7.9	4 – 35	T=6.182, P<0.001**	19.9	8.1	4 – 36	T=1.599, P=0.111	T=4.297, P<0.001**
**Post**	27.0	8.4	10 – 44		21.8	8.1	6 – 38		
**Student's behavior**									
**Pre**	16.5	6.5	4 – 30	T=5.703, P<0.001**	16.3	7.1	2 – 31	T=1.589, P=0.113	T=3.859, P<0.001**
**Post**	23.0	9.0	5 – 41		18.1	8.3	2 – 35		
**Student's attendance**									
**Pre**	16.8	7.6	2 – 32	T=7.656, P<0.001**	17.1	7.5	2 – 32	T=1.472, P=0.142	T=5.425, P<0.001**
**Post**	26.1	9.0	8 – 44		18.9	9.1	1 – 37		
**Total Students' academic achievement**									
**Pre**	89.0	20.0	49 – 129	T=13.826, P<0.001**	89.8	19.1	52 – 128	T=3.138, P=0.002*	T=11.516, P<0.001**
**Post**	128.2	18.7	91 – 166		98.1	16.9	64 – 132		

Note: Paired t-test (P1) = Comparison of each dimension of students' academic achievement among study group pre and post intervention.
Paired t-test (P2) = Comparison of each dimension of students' academic achievement among control group pre and post intervention.
Paired t-test (P3) = Comparison of each dimension of students' academic achievement study and control groups post intervention

The above table demonstrates the control groups, pre- and post-talent management educational interventions for the study, and descriptive statistics of students' academic achievement dimensions and their overall scores among the studied nursing students. As can be shown, at the pre-talent management educational intervention, the study and control groups' respective low total mean of academic attainment were (89.0 and 89.8). The table showed that the greatest mean score regarding students' academic achievement dimensions (academic performance (26.6), extracurricular activities (25.5), student interaction (27.0), student behavior (23.0), and the study group's post-intervention program saw a highly significant improvement in student attendance (26.1; $P < 0.0001$).
